# Double-lumen tubes verus single-lumen tube in patients undergoing minimally invasive cardiac surgery: a randomised, controlled clinical trial

**DOI:** 10.3389/fcvm.2025.1583360

**Published:** 2025-07-24

**Authors:** Zhenzhong Wang, Junfei Zhao, Yingjie Ke, Qiuji Wang, Yuxin Li, Yingxian Ye, Jianjun Zhang, Xiaogang Guo, Qingshi Zeng, Huanlei Huang

**Affiliations:** ^1^ Department of Cardiac Surgery, Guangdong Provincial People's Hospital (Guangdong Academy of Medical Sciences), Guangzhou, China; ^2^ Department of Anaesthesia, Guangdong Provincial People’s Hospital (Guangdong Academy of Medical Sciences), Guangzhou, China

**Keywords:** totally endoscopic cardiac valve surgery, endotracheal tube, hypoxemia, lung ventilation, postoperative complications

## Abstract

**Background:**

One-lung ventilation (OLV) with double-lumen tubes (DLT) are prone to complications such as airway injury and hypoxemia. It is not clear whether a two-lung ventilation (TLV) with single-lumen tube (SLT) is beneficial for patients undergoing totally endoscopic cardiac valve surgery (TECVS).

**Methods:**

We conducted a pragmatic, single-centre, single-blinded randomised controlled trial. Patients (aged ≥18 years) who underwent total endoscopic cardiac valve surgery were randomly assigned to a DLT group or a SLT group. A two-week telephone follow-up was conducted. The oxygenation index (PaO_2_/FiO_2_) was the primary outcome. The secondary outcomes included PaCO_2_, postoperative intubation complications, postoperative pulmonary complications and airway injury.

**Results:**

A total of 220 patients were randomly assigned. After randomisation, 20 patients were excluded, leaving 100 patients in each of the two groups. The PaO_2_/FiO_2_ were significantly greater in the SLT group than in the DLT group (*P* < 0.001). The incidence of postoperative intubation adverse events, postoperative pulmonary atelectasis, and hoarseness was significantly lower in the SLT group (*P* < 0.001, *P* = 0.029 and *P* = 0.028, respectively). The pre-exposure time and intubation time were shorter in the SLT group (both *P* values < 0.001). We used *t* test, Mann-Whitney *U* test and Fisher's exact test to account the difference of perioperative and follow-up outcomes.

**Conclusions:**

Two-lung ventilation with single-lumen tube is easy to perform, significantly increases oxygenation, and decreases the incidence of postoperative complications and airway injuries. Advantages remain especially for patients with preoperative pulmonary dysfunction.

**Clinical Trial Registration:**

https://www.chictr.org.cn/showproj.html?proj=165709, identifier [ChiCTR2200066822]. Date: 19/12/2022.

## Introduction

The use of thoracoscopic techniques in cardiac surgery began in the 1990s (1, 2). This approach requires one-lung ventilation (OLV) to achieve good field exposure. Double-lumen tube (DLT) being the most widely accepted modality today (3, 4).

It is well known that traditional DLTs not only require fibreoptic bronchoscopy positioning but are also prone to cause airway injury complications such as sore throat and hoarseness; moreover, in severe cases, airway tearing can occur (5–7). Even visualised DLTs do not reduce the incidence of airway injury (8). Thus, despite improvements in endotracheal tubes, it is still not possible to avoid the more severe airway injury, ventilation/blood flow mismatch, hypoxemia or postoperative pulmonary complications (PPCs) associated with OLV (9). For patients undergoing cardiac surgery, preoperative pulmonary congestion, pulmonary hypertension, cor pulmonale, and prolonged cardiopulmonary bypass further increase the risk. Although one-lung ventilation can indeed provide excellent surgical exposure, we believe it is not essential. This is particularly true given the current advancements in thoracoscopic techniques, where increasing attention is being paid to reducing postoperative pulmonary complications. The endotracheal diameter is 15–25 mm in adult males and 10–21 mm in adult females. The inner diameter of the DLT (Covidien, size #35–39, USA) is 4.8–5.3 mm, and the outer diameter of it is 11.7–13.0 mm. In contrast, the SLT (Covidien, size #6.0–8.0, USA) has a larger internal diameter (6.0–8.0 mm) and a thinner outer diameter (8.2–10.8 mm). The larger inner diameter of SLT indicates lower airflow resistance, while the thinner outer diameter tends to result in a lower incidence of airway injury. The advantages of a single-lumen tube (SLT) over a DLT are unquestionable. SLTs can achieve two-lung ventilation (TLV), which is more in line with the physiological state of normal respiration despite changing field exposure. There is no airway management model for TLV in TECVS. In our study, we used a lower tidal volume and a higher ventilation frequency to address this problem. Patients with preoperative combined moderate-severe pulmonary ventilatory dysfunction (PVD) may be ineligible for minimally invasive thoracoscopic surgery due to their inability to tolerate one-lung ventilation. Besides, this patient population is also at high risk for postoperative complications. Therefore, we included this group of patients in our clinical study in the hope of providing some evidence for airway management modalities in such patients.

The aim of this study was to clarify the effect of SLTs with TLV on oxygenation, postoperative airway injury, and pulmonary complications in thoracoscopic cardiac surgery, especially in patients with moderate-to-severe PVD.

## Methods

### Trial design

The study was a prospective, randomised, single-blinded, single-centre, controlled trial. Study period was from December 2021–April 2023. The trial was approved by the Medical Research Ethics Committee of Guangdong Provincial People's Hospital (KY-Q-2021-206-02). Written informed consent was provided by all study participants. Clinical trials are carried out in accordance with relevant laws and regulations and clinical trial protocols, and are subject to monitoring, verification and inspection. Investigators are trained in the experimental protocol prior to the start of the trial. All operations related to the trial are carried out in accordance with clinical trial management practices and related standard operations. The trial was registered in the Chinese clinical trial registry (ChiCTR2200066822, 19/12/2022).

### Eligibility criteria

Patients who met all of the following criteria were included: age ≥18 years, scheduling for elective TECVS, and agreement to participate. Potential subjects who met any of the following criteria were excluded: previous open-heart surgery or thoracic surgery, intraoperative conversion to median sternotomy, combined atrial septal defects or ventricular septal defects, pregnancy, airway stenosis, preoperative SaO_2_ < 90% or (and) PaO_2_ < 60 mmHg. In addition, we defined patients with moderate-to-severe PVD as high-risk patients.

### Randomisation, intervention, and follow-up

Using a randomisation list of permuted blocks that were computer-generated by an independent statistician, patients were randomly assigned to either the SLT group or the DLT group. Allocation concealment was achieved using sealed opaque envelopes. The patients are blind. Patients and study investigators assessing postoperative outcome parameters were both unaware of the randomisation result. Statistical analysis was performed blinded to study allocation.

To clarify the patients' immediate oxygenation status, arterial blood gas analyses (ABGA) were performed at the following five time points: T1: after successful endotracheal intubation and positioning; T2: 10 min of OLV or TLV before starting cardiopulmonary bypass (CPB); T3: half hour after the start of CPB; T4: 10 min of OLV or TLV after CPB was stopped; and T5: first blood gas analysis on admission to the intensive care unit.

All patients were operated on by the same professor. Tracheal intubation, anaesthesia induction, anaesthetic maintenance and resuscitation medicine were performed at the discretion of the attending anaesthesiologists. The anaesthesiologists are professional anesthesiologists who have worked for 5 years or more. In the SLT group, patients were intubated with an SLT (Covidien, size 6.0–8.0, USA). In the DLT group, patients were intubated using a DLT (Covidien, size Fr35–39, USA). The following parameters were consistent at T1 between the two groups: tidal volume (TV) 6–8 ml/kg, respiratory rate (RR) 12–20 beats/min, I:E = 1:2, positive end-expiratory pressure (PEEP) 5 mmHg, and FiO_2_ 60%. In the SLT group at T2, the following mode was used: TV 3 ml/kg, RR 30 beats/min, I:E = 1:2, PEEP 0 mmHg, and FiO_2_ 100%. In the DLT group, the following mode was used: TV 5 ml/kg, RR 17 beats/min, I:E = 1:2, PEEP 0 mmHg, and FiO_2_ 100%. CPB was performed in both groups at T3, FiO_2_ 50%. The management of intraoperative ventilation at T4 was the same as that at T2. The management of postoperative ventilation at T5 was the same as that at T1 except that the FiO_2_ was 80%. Because patients may not have been able to receive the intervention for 10 min at T2 and T4 due to hypoxemia, we decided that ABGA would be retained immediately when the patients' SpO_2_ was 85% with a tendency to decrease. Then, subsequent adjustments to the parameters of the ventilator or manipulation of the lungs for recovery were carried out to avoid the occurrence of severe hypoxemia, which could threaten the patients' lives.

All patients were followed up after 1 and 2 weeks by telephone interviews for complications including coughing, sore throat and hoarseness.

### Patient and public involvement statement

It was not appropriate or possible to involve patients or the public in the design, or conduct, or reporting, or dissemination plans of our research.

### Outcomes

The primary outcomes was the oxygenation index (OI, PaO_2_/FiO_2_).

The secondary outcomes included PaCO_2_, postoperative pulmonary complications (PPCs), airway injury, first intubation failure, tracheal tube displacement, poor visual field exposure and postoperative reintubation. For PPCs, we used the 2015 European definitions (10). Other definitions were as follows: (1) first intubation failure: unsuccessful first intubation with laryngoscopic assistance, with the end of the tracheal tube withdrawn above the vocal folds; (2) tracheal tube displacement: displacement observed by fibreoptic bronchoscopy or intraoperative discovery of a collapsed lung; (3) poor visual field exposure: brief cessation of the TLV due to obscuring of the visual field; and (4) postoperative reintubation: postoperative replacement of endotracheal tubes for any reason. Symptoms of airway injury included hoarseness, sore throat and cough.

### Statistical analysis

The postoperative PaO_2_/FiO_2_ ratio (mean ± standard deviation) was 326.9 ± 120.1 in the SLT group and 374.9 ± 130.9 mmHg in the DLT group in previous study (11). Sample estimation was performed by PASS version 15.0 with *α* = 0.05, power = 80%, and a 1:1 allocation ratio one-sided design, which suggested 86 patients per group. Assuming that approximately 10% of the patients withdrew or were lost to follow-up, each group was adjusted to 95 patients, for a total of 190 patients.

The Shapiro‒Wilk test was used to assess the normality of the data distribution. Continuous data with a normal distribution are presented herein as the mean ± standard deviation (SD), and comparisons between groups were made using the independent samples *t* test; continuous data with a nonnormal distribution are presented as the median (25th percentile, 75th percentile), and comparisons between groups were made using the Mann‒Whitney *U* test. Categorical data are presented as frequencies and percentages (%), and the chi-squared test or Fisher's exact test was used. All statistical analyses were conducted using SPSS 25.0, and a *P* value of less than 0.05 was considered to indicate statistical significance.

## Results

**Figure 1:**
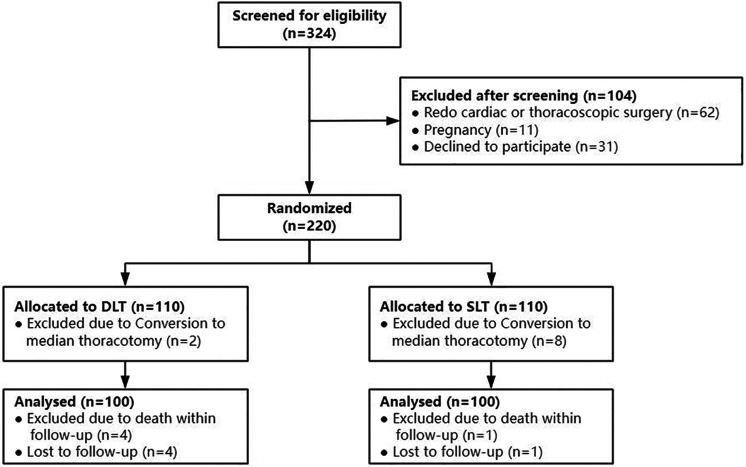
Flowchart of the included study participants. DLT, double-lumen tubes; SLT, single-lumen tube.

From December 2021 through April 2023, a total of 324 patients were screened for eligibility. After randomization, 20 patients (9.1% of the trial population) were excluded from the analysis, of which 10 cases were converted to median sternotomy, 5 cases were lost to follow-up, and 5 were death cases. One death occurred in the SLT group due to respiratory-circulatory failure. There were four deaths in the DLT group: three were due to left ventricular rupture, and one was due to postcardiac surgery low cardiac output syndrome combined with pulmonary infection. 5 patients (2.3%) lost to follow-up were not included for statistical analysis. Thus, a total of 200 patients were analysed (Figure 1). Each group consisted of 16 high-risk patients with moderate-to-severe PVD.

The patients' characteristics were similar between the groups (Table 1). The median intubation time in the DLT group was significantly longer [49 s (40–67 s) vs. 36 s (27–46 s), *P* < 0.001], as was the preexposure time [22 min (17–32 min) vs. 15 min (11–21 s), *P* < 0.001] (Table 2). The intubation time among high-risk patients was consistent with all patients [54 s (44–47 s) vs. 32 s (24–44 s), *P* = 0.006], but there was no significant difference in preexposure time (*P* = 0.941) (Table 2). There were no differences in CPB time, mechanical ventilation time or postoperative hospital stay (Table 2).

**Table 1:** Patient characteristics [mean ± SD/P50 (P25, P75)/%].

Characteristics	DLT group (*n* = 100)	SLT group (*n* = 100)	*P* value	High-Risk DLT group (*n* = 16)	High-Risk SLT group (*n* = 16)	*P* value
Sex			0.323			0.288
Male (%)	47	54	–	7	10	
Female (%)	53	46	–	9	6	
Age (years)	57 (46, 64)	56 (47, 61)	0.650	56 ± 9.4	54 ± 10.1	0.566
BMI (kg/m^2^)	23 ± 3.6	24 ± 3.5	0.199	24 ± 3.2	23 ± 3.5	0.858
ASA classification (%)			0.337			>0.999
Level II	12	6	–	1	0	
Level III	87	93	–	15	16	
Level IV	1	1	–	0	0	
Past medical history (%)						
Hypertension	24	24	>0.999	2	5	0.394
Diabetes (%)	5	6	>0.999	2	1	>0.999
CHD (%)	9	7	0.795	2	1	>0.999
Stroke (%)	3	8	0.213	1	0	>0.999
AF (%)	39	41	0.773	8	9	0.723
Smoking history (%)	15.0	15.0	>0.999	5	5	>0.999
Haemoglobin(g/L)	134 ± 18.1	136 ± 17.0	0.384	137 ± 17.8	137 ± 23.8	0.953
Haematocrit	0.413 ± 0.051	0.416 ± 0.048	0.655	0.419 ± 0.055	0.424 ± 0.068	0.787
Platelets (10^9^/L)	203 (173, 258)	202 (166, 238)	0.246	203 (165, 228)	195 (170, 239)	0.970
Albumin (g/L)	41 (39, 44)	41 (39, 44)	0.758	40 (36, 46)	41 (38, 42)	0.970
ALT (U/L)	21 (15, 31)	22 (15, 31)	0.417	19 (14, 31)	19 (14, 24)	0.720
Creatinine (µmol/L)	78 (63.9, 89.9)	78 (66.9, 95.8)	0.468	85 ± 26.3	86 ± 30.2	0.865
Preoperative LVDD (mm)	52 ± 7.4	52 ± 8.0	0.577	56 ± 8.9	55 ± 9.9	0.738
Preoperative LVEF (%)	65 (60, 69)	65 (61, 68)	0.706	61 ± 8.9	63 ± 7.4	0.638

BMI, body mass index; ASA, American society of anesthesiologists; CHD, coronary heart disease; AF, atrial fibrillation; ALT, alanine aminotransferase; LVDD, left ventricular diastolic dimension; LVEF, left ventricular ejection fraction.

**Table 2:** Perioperative variables [mean ± SD/P50 (P25, P75)/%].

Characteristics	DLT group (*n* = 100)	SLT group (*n* = 100)	*P* value	High-Risk DLT group (*n* = 16)	High-Risk SLT group (*n* = 16)	*P* value
Intubation time (s)^a^	49 (40, 67)	36 (27, 46)	<**0**.**001**	54 (44, 74)	32 (24, 44)	**0**.**006**
Preexposure time (min)^b^	22 (17, 32)	15 (11, 21)	<**0**.**001**	23 ± 10.1	23 ± 10.9	0.941
Postexposure time (min)^b^	30 (23, 40)	28 (25, 37)	0.678	34 ± 9.7	32 ± 11.6	0.527
CPB time (min)	165 (143, 201)	177 (137, 200)	0.740	202 ± 50.5	203 ± 45.3	0.933
ACC time (min)	103 (82, 129)	103 (80, 123)	0.308	130 ± 42.0	114 ± 28.2	0.202
POLS (d)	5 (3, 6)	4 (4, 5)	0.995	6 (4, 7)	5 (4, 7)	0.593
Mechanical ventilation time (min)	10 (4, 17)	10 (5, 17)	>0.999	15 (9, 21)	13 (7, 17)	0.485
24 h postoperative pleural fluid volume (ml)	185 (120, 276)	205 (140, 305)	0.198	286 ± 129.3	208 ± 137.3	0.107
ICU stay (h)	39 (21, 62)	40 (21, 47)	0.872	21 (40, 58)	46 (21, 85)	0.461
Postoperative LVDD (mm)	46 ± 6.0	47 ± 6.0	0.990	49 ± 5.7	50 ± 8.3	0.806
Postoperative LVEF (%)	60 (57, 64)	61 (58, 65)	0.334	61 (55, 64)	58 (53, 62)	0.130
Complications (%)						
Reoperation	1	0		0	0	-
Pericardial effusion	13	17	0.553	3	3	>0.999
Haemodialysis	0	2	0.497	0	0	-
IABP	2	1	>0.999	1	0	>0.999
ECMO	0	1	>0.999	0	0	-

CPB, cardiopulmonary bypass; ACC, aortic cross-clamp; POLS, postoperative length of stay; ICU, intensive care unit; LVDD, left ventricular diastolic dimension; LVEF, left ventricular ejection fraction; IABP, intraaortic balloon pump; ECMO, extracorporeal membrane oxygenation.

Bold values means *P* < 0.05.

^a^ Intubation time: Time taken to complete the endotracheal intubation, excluding the time for positioning.

^b^ Preexposure time: The time between chest incision and CPB initiation; Postexposure time: Time between CPB cessation and closing of the chest incision.

### Primary outcomes

**Figure 2:**
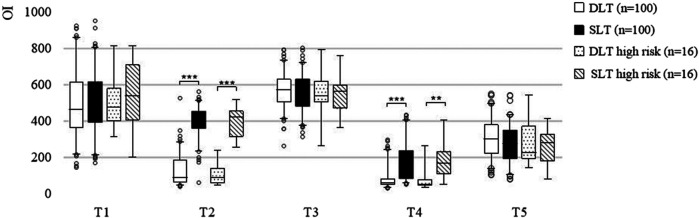
Nested box plot for the OIs of all patients and high-risk patients. OLV, one-lung ventilation; TLV, two-lung ventilation; T1–T5, described in the “Randomisation, Intervention and Follow-up” section. ***, *P* < 0.001; **, *P* < 0.01.

In the SLT group, the preoperative and postoperative OIs were significantly greater than those in the DLT group: 419 mmHg (359–456 mmHg) at T2 vs. 91 mmHg (61–187 mmHg) at T2 (*P* < 0.001) and 150 mmHg (83–240 mmHg) at T4 vs. 60 mmHg (49–86 mmHg) at T4 (*P* < 0.001) (Figure 2). Similar results were observed in high-risk patients. Oxygenation advantage of TLVs is significant regardless of the presence or absence of PVD.

### Secondary outcomes

**Figure 3:**
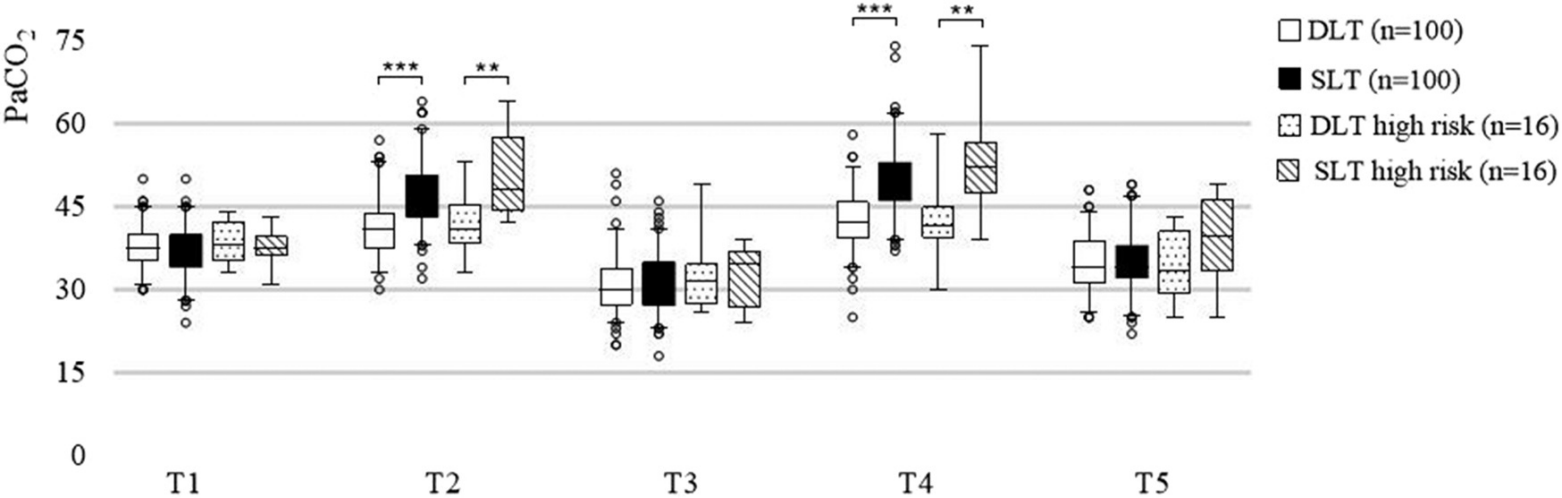
Nested box plot for the PaCO_2_ of all patients and high-risk patients. OLV, one-lung ventilation; TLV, two-lung ventilation; T1–T5, described in the “Randomisation, Intervention and Follow-up” section. ***, *P* < 0.001; **, *P* < 0.01.

The preoperative and postoperative PaCO_2_ were also greater in the SLT group: 47 mmHg (43–51 mmHg) at T2 vs. 41 mmHg (37–44 mmHg) at T2 (*P* < 0.001) and 50 mmHg (46–53 mmHg) at T4 vs. 42 mmHg (39–46 mmHg) at T4 (*P* < 0.001) (Figure 3). Similar results were observed in high-risk patients.

More first intubation failure were observed in the DLT group than in the SLT group (16% vs. 1%, *P* < 0.001). Visual field exposure was worse in the SLT group (2% vs. 10%, *P* = 0.017). All 6 cases of pulmonary atelectasis were observed in the DLT group (*P* = 0.029), with no significant difference in the other PPCs. Patients in the DLT group were more prone to hoarseness within one week after surgery (36% vs. 22%, *P* = 0.028), but there was no significant difference between the two groups within two weeks after surgery (*P* = 0.136). Subgroup analyses of high-risk patients revealed no apparent differences in the incidence of PPCs, or airway injury (Table 3).

**Table 3:** Secondary outcomes [mean ± SD/P50(P25, P75)/%].

Characteristics	DLT group (*n* = 100)	SLT group (*n* = 100)	*P* value	High-Risk DLT group (*n* = 16)	High-Risk SLT group (*n* = 16)	*P* value
First intubation failure (%)	16	1	**<0**.**001**	1	0	>0.999
Tracheal tube displacement (%)	2	0	0.497	0	0	–
Poor visual field exposure (%)	2	10	**0**.**017**	0	1	>0.999
Postoperative reintubation (%)	3	0	0.246	0	0	–
Postoperative pulmonary complications (%)						
Pleural effusion	53	63	0.197	10	12	0.704
Pulmonary exudation	68	67	>0.999	14	13	>0.999
Pneumothorax	9	4	0.251	2	0	0.484
Pneumonia	5	3	0.721	1	0	>0.999
Pulmonary atelectasis	6	0	**0**.**029**	3	0	0.226
Airway injury (%)						
Hoarseness within 1 week	36	22	**0**.**028**	4	3	>0.999
Sore throat within 1 week	12	9	0.645	0	2	0.484
Cough within 1 week	49	50	>0.999	6	9	0.479
Hoarseness within 2 weeks	22	13	0.136	3	2	>0.999
Sore throat within 2 weeks	3	3	>0.999	0	0	–
Cough within 2 weeks	28	21	0.324	4	4	>0.999

Bold values means *P* < 0.05.

## Discussion

TECVS is indicated in most patients with mitral and tricuspid valve disease and in a small percentage of patients with aortic valve (12). TECVS is often achieved by double lumen intubation and single lung ventilation. DLTs are frequently undersized or oversized, posing the risk of tube displacement or tracheal trauma (13). One-lung ventilation as an unphysiological method with disproportionate ventilation blood flow is prone to complications such as hypoxaemia and pulmonary atelectasis. It is not clear how single-lumen intubation with two-lung ventilation affects patient oxygenation and postoperative complications. In our randomised controlled trial of patients who received TECVS, compared with patients in the DLT group, patients in the SLT group not only had better oxygenation but also had shorter intubation times and significantly fewer cases of adverse intubation, postoperative pulmonary atelectasis, and hoarseness. However, compared to the DLT group, the SLT group was also prone to hypercapnia and had inferior visual field exposure.

To protect the patient's lung function, the OLV parameters were set with reference to the protective ventilation strategy, which mainly consisted of low tidal volumes and PEEP (14). In order to avoid the effect of PEEP on oxygenation and lung collapse, PEEP was not set in this study. Low tidal volumes (4–6 ml/kg) during OLV have been shown to reduce the incidence of acute respiratory distress syndrome, reduce pulmonary infiltrates and promote oxygenation (15). To maintain a balance of tidal volumes between the two groups, we experimentally used a tidal volume of 3 ml/kg with an increase in respiratory rate and found that this setting was not prone to cause hypoxia, in agreement with the results of the T2 blood gas analysis.

The incidence of hypoxemia in thoracoscopic surgery with OLV is approximately 6.5%–18% (16–18). Many attempts have been made by researchers to improve patients' oxygenation during OLV, such as giving ventilation on the collapsed lung (low tidal volume of 1–2 ml/kg and high frequency of 40 times/min) or applying continuous positive airway pressure(5 cm H_2_O), and the results have shown that oxygenation is superior to OLV (19, 20). Our trial showed that the preoperative and postoperative OI values were greater in the SLT group than in the DLT group, and more importantly, the OI value at T2 in our study [419 (359,456)] was also greater than that in the other clinical studies mentioned above. This further demonstrates the significant superiority of the TLV mode in oxygenation. The reason is TLV has a better sustained ventilation/blood flow ratio compared to OLV. Moreover, TLV is more simple to perform for anaesthesiologist than other method.

TLV also caused mild-to-moderate hypercapnia in patients [PaCO_2_: 47(43,51) mmHg at T2, 50(46,53) mmHg at T4]. Although the total tidal volumes is consistent, the tidal volume and exhalation time are both less per ventilation during TLV compared to OLV. This is unfavorable for carbon dioxide (CO_2_) elimination, making it easier for CO_2_ to accumulate in the lungs. Additionally, CO_2_ is highly soluble in blood, which further contributes to more significant hypercapnia in TLV patients. Hypercapnia is associated with adverse effects such as increased pulmonary artery pressure (21), but this risk is acceptable, based on our findings. First, the duration of hypercapnia is short and easily modifiable. Hypercapnia began to appear only in the middle and late stages of TLV. The anaesthesiologist would adjust the ventilator parameters to improve hypercapnia immediately after collecting the sample for blood gas analysis sample. Second, hypercapnia has some benefits. Mild hypercapnia increases cardiac output, enhances myocardial contractility, reduces afterload (22) and is beneficial for increasing the local oxygen supply to the brain (23). Moderate hypercapnia (60–70 mmHg) has been shown to significantly increase cardiac output and the cardiac index in patients undergoing open thoracic pulmonary resection and is tolerated by patients (24). Third, in our study, hypercapnia did not prolong postoperative mechanical ventilation (*P* = 0.946). Taken together, the benefits of improved oxygenation produced by high-frequency low-tidal-volume TLV outweigh the adverse effects of hypercapnia.

Differences in the mode of ventilation also imply differences in the mode of intubation, which also leads to differences in visual field exposure. The incidence of first intubation failure was significantly greater in the DLT group than in the SLT group (16% vs. 1%, *P* < 0.001) (Table 3) while the incidence of poor visual field exposure was lower in the DLT group (2% vs. 10%, *P* = 0.017). Although the visual field exposure was inferior to that in the DLT group, a good surgical visual field was obtained by starting CPB earlier [preexposure time: 15 (11, 21) min vs. 22 (17, 32) min, *P* < 0.001] and did not significantly prolong the total CPB time in the SLT group (*P* = 0.740) (Table 2). Notably, the intubation time was significantly longer in the DLT group (*P* < 0.001) (Table 2). This suggests that the advantages of one-lung ventilation in terms of visual field exposure did not expedite surgical execution, but rather introduced additional complications due to the difficulty of manipulating and positioning the double-lumen intubation. Based on the thinner outer diameter and less first intubation failure, SLT is particularly suitable for thin women or those with narrow airways. Thus, with OLV, convenience and oxygenation were sacrificed for good visual field exposure, whereas a better balance was struck with TLV.

The incidence of PPCs after cardiac surgery is approximately 19%–69%, and PPCs are associated with increased mortality, length of stay in the intensive care unit (ICU) and length of hospital stay (25–29). In our study, the most common PPC was pulmonary exudation, with an overall incidence of 68%. We believe that postoperative pulmonary exudation might be related to impaired surface-active substances and increased airway secretions and can be improved by bronchodilators and sputum elimination. Notably, all cases of postoperative lung atelectasis were observed in the DLT group (6% vs. 0%, *P* = 0.029), and all atelectasis occurred in the right lung, i.e., the collapsed side of the lung. We believe that the main reasons for this finding were as follows: (1) prolonged lung collapse leading to low alveolar pressure and an imbalance of pressure between inside and outside the alveoli; and (2) a low ventilation/blood flow ratio of the collapsed side of the lung, which is more likely to lead to absorptive lung atelectasis. Postoperative pulmonary atelectasis is closely associated with other complications, such as hypoxaemia, atelectasis-associated pneumonia and respiratory failure, which prolong the length of hospital stay and mechanical ventilation. Therefore, TLV is beneficial for reducing pulmonary atelectasis.

We have clarified that TLV increases oxygenation and decreases postoperative intubation and pulmonary complications, but the differences in airway injury between the two groups are unclear. Clinical symptoms of airway injury mainly include hoarseness, coughing up sputum, pain, and choking. The incidence of hoarseness within 7 days postoperatively was significantly higher in the DLT group than in the SLT group in our study (36% vs. 22%, *P* = 0.028). In previous clinical studies, postoperative hoarseness was not uncommon in patients receiving DLT, with an incidence ranging from 10%–47.4%, which was higher than that in patients receiving bronchial blockers (0%–27.8%) or laryngeal masks (3%) (5, 30–32). Such hoarseness is caused mainly by mechanical damage to the vocal cords during intubation and extubation. Compared with SLTs, DLTs are more prone to cause mechanical damage because of their greater diameter and longer intubation time (*P* < 0.001). It is worth noting that only one patient still experienced choking while drinking water 15 days after the operation, and this symptom did not disappear until 2 months after the operation; this patient is now considered to have recurrent laryngeal nerve injury. Although this event is less common, the recovery time for patients with nerve damage is long, and nerve damage has a significant adverse effect on their quality of life. In sum, SLTs are more advantageous in terms of airway damage and manipulation.

Importantly, we extended the trial evidence to the high-risk population with moderate-to-severe PVD, which has previously been largely excluded from clinical trials, but it increases operative mortality (33). Moderate-to-severe PVD is present in 21% of patients before cardiac surgery (34). There is no definitively recommended mode of intubation or ventilatory management for these patients. Consistent with previous results, both preoperative and postoperative oxygenation were significantly better in the SLT group than in the DLT group among high-risk patients, while hypercapnia also persisted, and there was no significant difference in postoperative complications between the two groups. The hypercapnia does not prolong the length of hospital stay or duration of mechanical ventilation. Our results suggest that patients with moderate-to-severe PVD will not be ineligible for minimally invasive cardiac surgery or forced to undergo median sternotomy cardiac surgery because they cannot tolerate OLV.

The limitation of this study is that hypoxaemia caused a proportion of patients to be unable to receive the ten-minute intervention. This occurred in the DLT group is 19%. It is clear that as time increases, the patient's partial pressure of oxygen becomes lower as well as the partial pressure of carbon dioxide higher. Therefore, this does not change our conclusions, rather it confirms our conclusions from the side. In addition to this, we did not investigate the effect of more ventilation parameters on perioperative outcomes, such as positive end-expiratory pressure, and more clinical data is needed.

## Conclusion

We suggest that two-lung ventilation can be used in thoracoscopic cardiac surgery because of its better performance in terms of oxygenation, postoperative horseness, and postoperative pulmonary atelectasis. Surgeons with little experience in totally thoracoscopic cardiac surgery should consider the adverse effects of it on visual field exposure.

## Data Availability

The original contributions presented in the study are included in the article/Supplementary Material, further inquiries can be directed to the corresponding author.

## References

[1] Chitwood WR Wixon CL Elbeery JR Francalancia NA Lust RM. Minimally invasive cardiac operation: adapting cardioprotective strategies. Ann Thorac Surg. (1999) 68:1974–7. 10.1016/S0003-4975(99)01019-X 10585114

[2] Laborde F Noirhomme P Karam J Batisse A Bourel P Maurice OS. A new video-assisted thoracoscopic surgical technique for interruption of patient ductus arteriosus in infants and children. J Thorac Cardiovasc Surg. (1993) 105:278–80. 10.1016/S0022-5223(19)33812-7 8429655

[3] Brodsky JB Lemmens HJ. Left double-lumen tubes: clinical experience with 1,170 patients. J Cardiothorac Vasc Anesth. (2003) 17:289–98. 10.1016/S1053-0770(03)00046-6 12827573

[4] Ehrenfeld JM Walsh JL Sandberg WS. Right- and left-sided Mallinckrodt double-lumen tubes have identical clinical performance. Anesth Analg. (2008) 106:1847–52. 10.1213/ane.0b013e31816f24d5 18499621

[5] Risse J Szeder K Schubert AK Wiesmann T Dinges HC Feldmann C Comparison of left double lumen tube and y-shaped and double-ended bronchial blocker for one lung ventilation in thoracic surgery-a randomised controlled clinical trial. BMC Anesthesiol. (2022) 22:92. 10.1186/s12871-022-01637-1 35366801 PMC8976407

[6] Knoll H Ziegeler S Schreiber JU Buchinger H Bialas P Semyonov K Airway injuries after one-lung ventilation: a comparison between double-lumen tube and endobronchial blocker: a randomized, prospective, controlled trial. Anesthesiology. (2006) 105:471–7. 10.1097/00000542-200609000-00009 16931978

[7] Tezel C Okur E Baysungur V. Iatrogenic tracheal rupture during intubation with a double-lumen tube. Thorac Cardiovasc Surg. (2010) 58:54–6. 10.1055/s-0029-1186150 20072981

[8] Palaczynski P Misiolek H Bialka S Owczarek AJ Gola W Szarpak Ł A randomized comparison between the VivaSight double-lumen tube and standard double-lumen tube intubation in thoracic surgery patients. J Thorac Dis. (2022) 14:3903–14. 10.21037/jtd-22-451 36389329 PMC9641341

[9] Purohit A Bhargava S Mangal V Parashar VK. Lung isolation, one-lung ventilation and hypoxaemia during lung isolation. Indian J Anaesth. (2015) 59:606–17. 10.4103/0019-5049.165855 26556920 PMC4613408

[10] Jammer IB Wickboldt N Sander M Smith A Schultz MJ Pelosi P Standards for definitions and use of outcome measures for clinical effectiveness research in perioperative medicine: European perioperative clinical outcome (EPCO) definitions: a statement from the ESA-ESICM joint taskforce on perioperative outcome measures. Eur J Anaesthesiol. (2015) 32:88–105. 10.1097/EJA.0000000000000118 25058504

[11] Kang WS Yoon TG Kim TY Kim SH. Comparison of the PaO_2_/FiO_2_ ratio in sternotomy vs. thoracotomy in mitral valve repair: a randomised controlled trial. Eur J Anaesthesiol. (2011) 28:807–12. 10.1097/EJA.0b013e32834ad993 21897262

[12] Gu W Zhou K Wang Z Zang X Guo H Gao Q Totally endoscopic aortic valve replacement: techniques and early results. Front Cardiovasc Med. (2023) 9:1106845. 10.3389/fcvm.2022.1106845 36698939 PMC9868623

[13] Roldi E Inghileri P Dransart-Raye O Mongodi S Guinot PG Mojoli F Use of tracheal ultrasound combined with clinical parameters to select left double-lumen tube size: a prospective observational study. Eur J Anaesthesiol. (2019) 36:215–20. 10.1097/EJA.0000000000000939 30540641

[14] Futier E Constantin JM Paugam-Burtz C Pascal J Eurin M Neuschwander A A trial of intraoperative low-tidal-volume ventilation in abdominal surgery. N Engl J Med. (2013) 369:428–37. 10.1056/NEJMoa1301082 23902482

[15] Campos JH Feider A. Hypoxia during one-lung ventilation-a review and update. J Cardiothorac Vasc Anesth. (2018) 32:2330–8. 10.1053/j.jvca.2017.12.026 29361458

[16] Wang W Gong Z Zhao M Zhang Z Qiu Y Jiang Q Hypoxemia in thoracoscopic lung resection surgery using a video double-lumen tube versus a conventional double-lumen tube: a propensity score-matched analysis. Front Surg. (2023) 10:1090233. 10.3389/fsurg.2023.1090233 36874459 PMC9982010

[17] Lu Y Dai W Zong Z Xiao Y Wu D Liu X Bronchial blocker versus left double-lumen endotracheal tube for one-lung ventilation in right video-assisted thoracoscopic surgery. J Cardiothorac Vasc Anesth. (2018) 32:297–301. 10.1053/j.jvca.2017.07.026 29249583

[18] Jung DM Ahn HJ Jung SH Yang M Kim JA Shin SM Apneic oxygen insufflation decreases the incidence of hypoxemia during one-lung ventilation in open and thoracoscopic pulmonary lobectomy: a randomized controlled trial. J Thorac Cardiovasc Surg. (2017) 154:360–6. 10.1016/j.jtcvs.2017.02.054 28412111

[19] Kremer R Aboud W Haberfeld O Armali M Barak M. Differential lung ventilation for increased oxygenation during one lung ventilation for video assisted lung surgery. J Cardiothorac Surg. (2019) 14:89. 10.1186/s13019-019-0910-2 31060627 PMC6503433

[20] Feng Y Wang J Zhang Y Wang S. One-lung ventilation with additional ipsilateral ventilation of low tidal volume and high frequency in lung lobectomy. Med Sci Monit. (2016) 22:1589–92. 10.12659/MSM.895294 27166086 PMC4913818

[21] Chan MJ Lucchetta L Cutuli S Eyeington C Glassford NJ Mårtensson J A pilot randomized controlled study of mild hypercapnia during cardiac surgery with cardiopulmonary bypass. J Cardiothorac Vasc Anesth. (2019) 33:2968–78. 10.1053/j.jvca.2019.03.012 31072710

[22] Almanza-Hurtado A Guerra CP Martínez-Ávila MC Borré-Naranjo D Rodríguez-Yanez T Dueñas-Castell C. Hypercapnia from physiology to practice. Int J Clin Pract. (2022) 2022:2635616. 10.1155/2022/2635616 36225533 PMC9525762

[23] Wong C Churilov L Cowie D Tan CO Hu R Tremewen D Randomised controlled trial to investigate the relationship between mild hypercapnia and cerebral oxygen saturation in patients undergoing major surgery. BMJ Open. (2020) 10:e029159. 10.1136/bmjopen-2019-029159 32066598 PMC7045198

[24] Masterson C Horie S McCarthy SD Gonzalez H Byrnes D Brady J Hypercapnia in the critically ill: insights from the bench to the bedside. Interface Focus. (2021) 11:20200032. 10.1098/rsfs.2020.0032 33628425 PMC7898152

[25] Li XF Jiang RJ Mao WJ Yu H Xin J Yu H. The effect of driving pressure-guided versus conventional mechanical ventilation strategy on pulmonary complications following on-pump cardiac surgery: a randomized clinical trial. J Clin Anesth. (2023) 89:111150. 10.1016/j.jclinane.2023.111150 37307653

[26] Mathis MR Duggal NM Likosky DS Haft JW Douville NJ Vaughn MT Intraoperative mechanical ventilation and postoperative pulmonary complications after cardiac surgery. Anesthesiology. (2019) 131:1046–62. 10.1097/ALN.0000000000002909 31403976 PMC6800803

[27] Ball L Volta CA Saglietti F Spadaro S Di Lullo A De Simone G Associations between expiratory flow limitation and postoperative pulmonary complications in patients undergoing cardiac surgery. J Cardiothorac Vasc Anesth. (2022) 36:815–24. 10.1053/j.jvca.2021.07.035 34404594

[28] Mohamed MA Cheng C Wei X. Incidence of postoperative pulmonary complications in patients undergoing minimally invasive versus median sternotomy valve surgery: propensity score matching. J Cardiothorac Surg. (2021) 16:287. 10.1186/s13019-021-01669-7 34627311 PMC8501915

[29] Cavayas YA Eljaiek R Rodrigue É Lamarche Y Girard M Wang HT Preoperative diaphragm function is associated with postoperative pulmonary complications after cardiac surgery. Crit Care Med. (2019) 47:e966–74. 10.1097/CCM.0000000000004027 31609771

[30] Huang C Huang Q Shen Y Liu K Wu J. General anaesthesia with double-lumen intubation compared to opioid-sparing strategies with laryngeal mask for thoracoscopic surgery: a randomised trial. Anaesth Crit Care Pain Med. (2022) 41:101083. 10.1016/j.accpm.2022.101083 35472588

[31] Palaczynski P Misiolek H Szarpak L Smereka J Pruc M Rydel M Systematic review and meta-analysis of efficiency and safety of double-lumen tube and bronchial blocker for one-lung ventilation. J Clin Med. (2023) 12:1877. 10.3390/jcm12051877 36902663 PMC10003923

[32] Liu Z Zhao L Zhu Y Bao L Jia QQ Yang XC The efficacy and adverse effects of the Uniblocker and left-side double-lumen tube for one-lung ventilation under the guidance of chest CT. Exp Ther Med. (2020) 19:2751–6. 10.3892/etm.2020.8492 32256757 PMC7086194

[33] Adabag AS Wassif HS Rice K Mithani S Johnson D Bonawitz-Conlin J Preoperative pulmonary function and mortality after cardiac surgery. Am Heart J. (2010) 159:691–7. 10.1016/j.ahj.2009.12.039 20362731

[34] Henn MC Zajarias A Lindman BR Greenberg JW Melby SJ Quader N Preoperative pulmonary function tests predict mortality after surgical or transcatheter aortic valve replacement. J Thorac Cardiovasc Surg. (2016) 151:578–86. 10.1016/j.jtcvs.2015.10.067 26687886 PMC5091079

