# Supplementary material for: Double-lumen tubes verus single-lumen tube in patients undergoing minimally invasive cardiac surgery: a randomised, controlled clinical trial
Source: Front Cardiovasc Med. 2025 Jul 24;12:1583360. doi: 10.3389/fcvm.2025.1583360 (PMC12328442; doi:10.3389/fcvm.2025.1583360)
Supplement: Supplementary file 1 [file Image1.pdf]

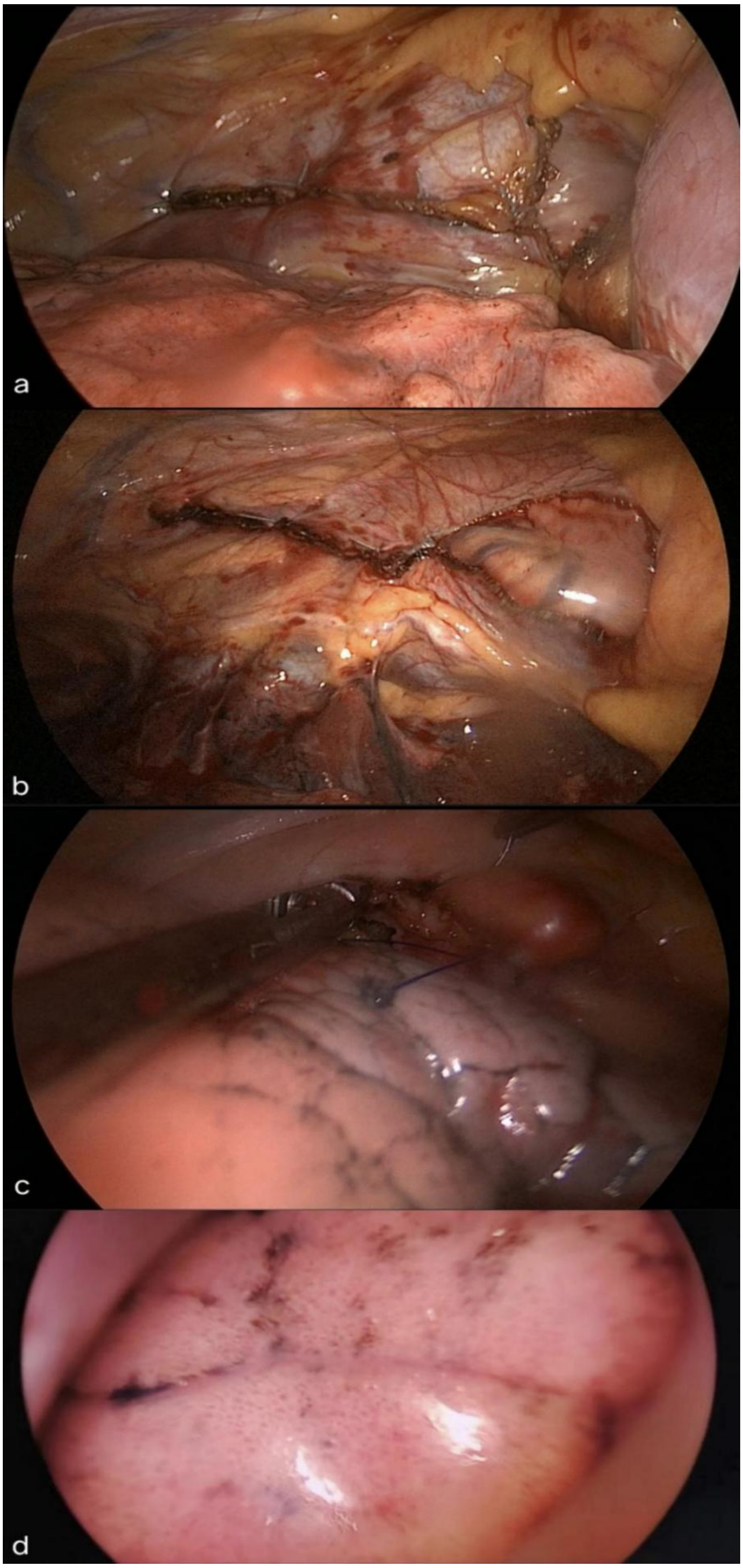

Supplementary Figure 1. This is the figure of thoracoscopic exposure of the operative field:

a: good postoperative exposure in the SLT group;

b: good postoperative exposure in the DLT group;
c: poor postoperative exposure in the SLT group;
d: .postoperative endotracheal tube displacement in the DLT group.
